# The effects of resveratrol on lipid profiles and liver enzymes in patients with metabolic syndrome and related disorders: a systematic review and meta-analysis of randomized controlled trials

**DOI:** 10.1186/s12944-020-1198-x

**Published:** 2020-02-17

**Authors:** Maryam Akbari, Omid Reza Tamtaji, Kamran B. Lankarani, Reza Tabrizi, Ehsan Dadgostar, Neda Haghighat, Fariba Kolahdooz, Amir Ghaderi, Mohammad Ali Mansournia, Zatollah Asemi

**Affiliations:** 1 Health Policy Research Center, Institute of Health, Student Research Committee, Shiraz University of Medical Sciences, Shiraz, Iran; 2 Research Center for Biochemistry and Nutrition in Metabolic Diseases, Kashan University of Medical Sciences, Kashan, IR Iran; 3 Health Policy Research Center, Shiraz University of Medical Sciences, Shiraz, Iran; 4 Halal Research Center of IRI, FDA, Tehran, Iran; 5 Nutrition Research Center, School of Nutrition and Food Sciences, Shiraz University of Medical Sciences, Shiraz, Iran; 6 Indigenous and Global Health Research, Department of Medicine, University of Alberta, Edmonton, Canada; 7 Department of Addiction studies, School of Medical, Kashan University of Medical Sciences, Kashan, Iran; 8 Clinical Research Development Unit-Matini/Kargarnejad Hospital, Kashan University of Medical Sciences, Kashan, IR Iran; 9 Department of Epidemiology and Biostatistics, School of Public Health, Tehran University of Medical Sciences, Tehran, Iran

**Keywords:** Resveratrol, Lipid profiles, Liver enzymes, Metabolic syndrome, Meta-analysis

## Abstract

**Background:**

There are current trials investigating the effect of resveratrol supplementation on lipid profiles and liver enzymes among patients with metabolic syndrome (MetS) and related disorders; however, their findings are controversial. This systematic review and meta-analysis were aimed to determine the effects of resveratrol supplementation on lipid profiles and liver enzymes among patients with MetS and related disorders.

**Methods:**

We performed a comprehensive search of the following online databases up to November 2018: Cochrane Library, PubMed, Embase, and Web of Science. The relevant articles were assessed for quality of studies using the Cochrane risk of bias tool.

**Results:**

Out of 2459 citations, 31 articles were appropriate for including to the current meta-analysis. The pooled results indicated that resveratrol use significantly decreased total cholesterol [weighted mean difference (WMD) = − 7.65 mg/dL; 95% CI, − 12.93, − 2.37; *P* < 0.01; I^2^: 83.4%] and increased gamma-glutamyl transferase (GGT) concentrations (WMD = 1.76 U/l; 95% CI, 0.58, 2.94; *P* < 0.01; I^2^: 20.1%). We found no significant effect of resveratrol supplementation on triglycerides (WMD = − 5.84 mg/dL; 95% CI, − 12.68, 1.00; *P* = 0.09; I^2^: 66.8%), LDL- (WMD = -2.90 mg/dL; 95% CI, − 10.88, 5.09; *P* = 0.47; I^2^: 96.0%), HDL-cholesterol (WMD = 0.49 mg/dL; 95% CI, − 0.80, 1.78; *P* = 0.45; I^2^: 74.0%), alanine aminotransferase (ALT) (WMD = -0.14 U/l; 95% CI, − 3.69, 3.41; *P* = 0.93; I^2^: 79.6%), and aspartate aminotransferase (AST) (WMD = -0.34 U/l; 95% CI, − 2.94, 2.27; *P* = 0.80; I^2^: 88.0%) concentrations.

**Conclusions:**

This meta-analysis demonstrated that resveratrol supplementation among patients with MetS and related disorders significantly reduced total cholesterol and increased GGT concentrations, but did not affect triglycerides, LDL-, HDL-cholesterol, ALT, and AST concentrations. This data suggests that resveratrol may have a potential cardio-protective effect in patients with MetS and related disorders.

## Background

Increased concentrations of circulating lipid profiles are a strong risk factor for cardiovascular disease [1]; high concentrations of total-, LDL-cholesterol, or triglycerides, as well as, low concentrations of HDL-cholesterol are consistently correlated with incidence of cardiovascular diseases (CVDs) [2, 3]. Metabolic syndrome (MetS) is considered as an insulin resistant syndrome comprising impaired glucose tolerance, decreased insulin sensitivity, dyslipidemia, central obesity, and hypertension, all of which are well- established risk factors for CVDs [4]. In addition, MetS is correlated with non-alcoholic fatty liver disease (NAFLD), type 2 diabetes mellitus (T2DM), colorectal disease, atrial fibrillation and hypothyroidism [5, 6]. NAFLD is also associated with impaired liver enzymes, including, alanine aminotransferase (ALT) and aspartate aminotransferase (AST), dysfunctional fat cells, and adipose tissue insulin resistance, resulting in hyperglycemia and dyslipidemia [7, 8].

The beneficial effects of resveratrol, plant sterols, and stanols on lipid profiles and modifying cardiovascular risk factors have been reported [9–13]. Resveratrol is a natural polyphenolic compound found mainly in peanuts and in the skin of red grapes that is used as a dietary supplement to improve metabolic profiles [14]. The effects of resveratrol supplementation on lipid profiles and liver enzymes have already been evaluated; however, these findings are controversial. In a meta-analysis on seven randomized controlled trials (RCTs), conducted by Sahebkar et al. [15], resveratrol supplementation had no effect on lipid profile. In another meta-analysis conducted by Hausenblas et al. [16], resveratrol supplementation to patients with T2DM was more effective on the systolic blood pressure, hemoglobin A1c, and creatinine, but did not affect fasting glucose, insulin resistance, diastolic blood pressure, insulin, triglycerides, LDL-and HDL-cholesterol concentrations. However, another meta-analysis of then RCTs showed no effects of resveratrol supplementation on total-, LDL-cholesterol, triglycerides, and fasting glucose concentrations [17]. Differences in study design, study population’s characteristics, the dosage of resveratrol used, and the duration of intervention might explain the discrepancies among different studies.

We aimed to systematically review the trials investigating the effect of resveratrol supplementation on lipid profiles and liver enzymes and to summarize the impact among patients with MetS and related disorders.

## Methods

PRISMA guideline (ERF) (the preferred reporting items for systematic reviews and meta-analyses) was used to design and implement this meta-analysis.

### Search strategy

Two independent authors (MA and OT) performed a comprehensive search to identify the relevant RCTs through inception up to November 2018. Online databases, including Cochrane Library, PubMed, Embase, and Web of Science databases by using the following MeSH and text keywords: patients [“Mets” OR “NAFLD” OR “disorders related to MetS” OR “diabetes” OR “T1DM” OR “T2DM” OR “overweight” OR “obese” OR “chronic kidney disease” OR “hypertension” OR “high blood pressure” OR “dyslipidemia” OR “CVD”], intervention (“resveratrols” OR “resveratrol” AND “use” OR “supplementation” OR “intake”), and outcomes lipid profiles [“triglycerides” OR “total cholesterol” OR “LDL-cholesterol (LDL-C)” OR “HDL-cholesterol (HDL-C)”] and liver measurements [“alanine aminotransferase (ALT)” OR “aspartate aminotransferase (AST)” OR “gamma-glutamyl transferase (GGT)”]. Clinical trials retrieved that estimated the effect of resveratrol intake on lipid profiles and/or liver enzymes. Our search strategy was limited to human RCTs published in English language. We conducted a manual search in the reference list’s included articles and pervious relevant reviews to find other additional articles.

### Selection criteria

The following inclusion criteria were used to select the related articles: RCTs were among humans (with parallel or cross-over design) with metabolic diseases, administrated resveratrol supplements in the intervention group and received placebo in the comparison group, contained sufficient data on mean changes of lipid profiles (including, triglycerides, total-, LDL-, and HDL-cholesterol concentrations), and liver enzymes (ALT, AST, and GGT concentrations), along with standard deviation (SD) or related 95% confidence intervals (CIs) at the baseline and at the end of trial for the intervention and placebo groups. RCTs which were not placebo controlled or other type of studies including animal, in vitro, case report, and case series, also abstracts or protocols without full texts, and studies with dosage of resveratrol lower than 20 mg/day were excluded.

### Data extraction

Two independent investigators (MA and OT) extracted data using a standard Excel forms according to the following items: first author’s name, publication year, country, demographic characteristics of participants, study methods, sample size (intervention/placebo groups), dose of treatment, type of intervention, type of diseases, the mean ± (SD) of changes for triglycerides, total-, LDL-, HDL-cholesterol, ALT, AST, and GGT concentrations in the intervention and placebo groups at the baseline and at the end of intervention. If the outcomes were reported by different doses, types of supplements, or duration of the intervention, we treated each situation as a separate study. Disagreements were resolved by discussion with a third author (ZA).

### Quality assessment

The Cochrane Collaboration risk of bias tool was applied to assess the quality of selected RCTs using the following domains: “randomization generation, allocation concealment, blinding of participants and outcome assessment, incomplete outcome data and selective outcome reporting, and the other sources of bias”.

### Statistical analysis

All statistical analyses were conducted using STATA software version 12.0 (Stata Corp., College Station, TX) and RevMan V.5.3 software (Cochrane Collaboration, Oxford, UK). Weighted mean differences (WMDs) and 95% CIs were considered as the overall combined effect sizes. Heterogeneity across included trials was examined using the Cochrane’s Q and I^2^ statistics. I^2^ > 50% with *P* < 0.05 indicated that a significant heterogeneity exists, therefore, the DerSimonian and Laird random effects model were used to combine effect sizes; otherwise, the inverse variance fixed-effect model was applied. Sensitivity analyses were performed to evaluate the impact of each included clinical trials on the validity of the overall combined WMDs. Subgroup analyses were conducted to examine the source of heterogeneity according to the following possible moderator variables; type of interventions (resveratrol plus other nutrients or drugs vs. resveratrol alone), dosage of resveratrol (> 250 vs. ≤250 mg/day), duration of intervention (≤8 vs. > 8 weeks), and type of chronic condition (e.g. overweight, or obese, or other chronic diseases vs. T2DM). The potential evidence of publication bias was determined using Egger’s- and Begg’s-test. *P*-value less than 0.05 were considered as statistically significant.

## Results

**Fig. 1:**
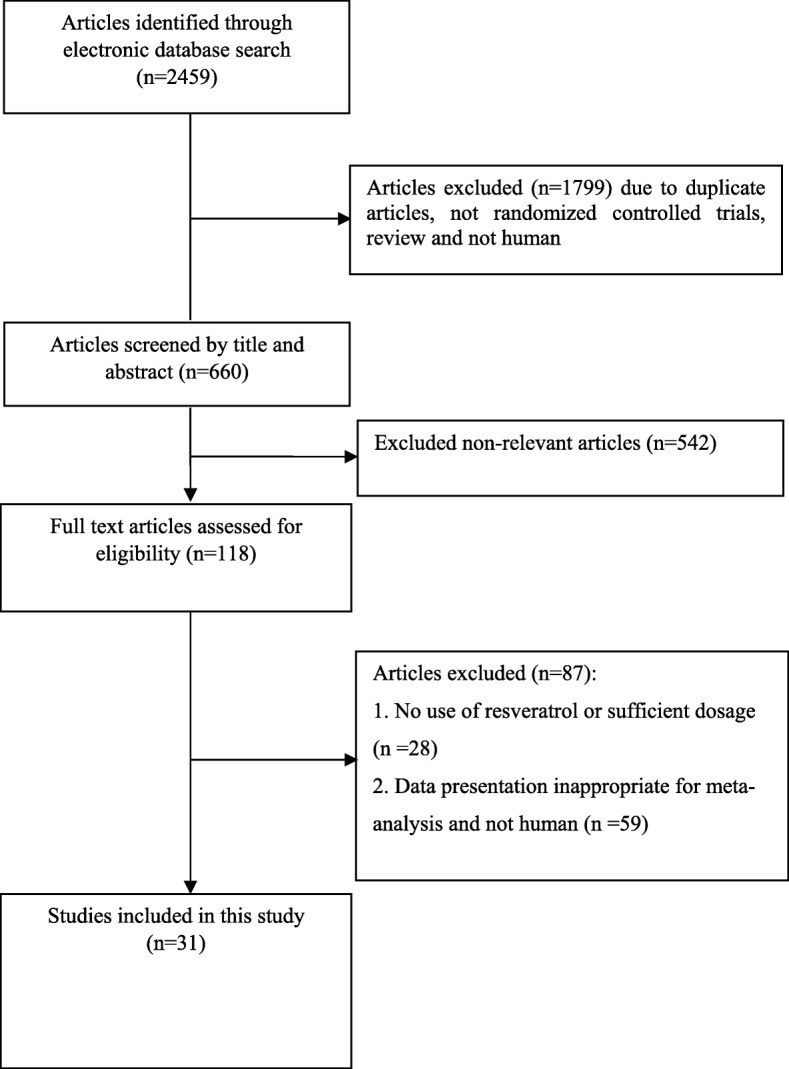
Literature search and review flowchart for selection of studies

**Fig. 2:**
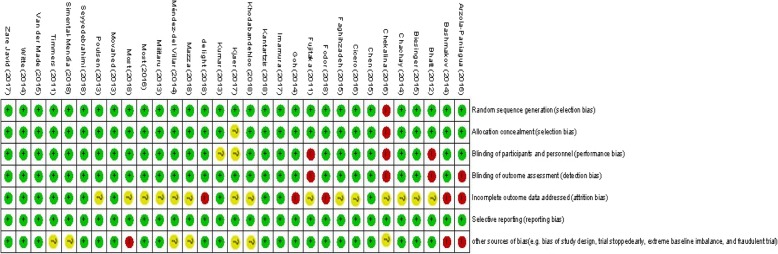
The summary of review authors’ judgments about each risk of bias item for each included study

In initial online database searches, 2459 reports were identified. After removing duplicates citations by reviewing titles and abstracts and excluding the irrelevant citations, 31 studies (35 effect sizes) were finally included. Figure 1 shows the stepwise with more details of the identification and selection of the relevant articles. All 35 included effect sizes were randomized, placebo-controlled trial. Twenty-nine studies were conducted using parallel design and six studies had cross-over design. The total number of the participants among included studies was 1722 individuals (890 persons in the resveratrol group; and 832 in the placebo group). Thirty-two studies calculated the influences of resveratrol intake on triglycerides, twenty-eighth on total cholesterol, twenty-seven on LDL-cholesterol, twenty-nine on HDL-cholesterol, thirteen on ALT, ten on AST, and five studies on GGT concentrations. The duration of resveratrol supplements ranged from four to 48 weeks and dosage of the intervention varied from 20 to 3000 mg/day among included articles. Table 1 illustrates the characteristics of the included articles. The quality assessment of included articles performed by authors’ judgment according to each bias item is presented in Fig. 2**.**

**Table 1:** Characteristics of included studies

Authors (Ref)	Publication year	Sample size (control/intervention)	Population/Country	Intervention (name and daily dose)	Duration	Presented data	Age (y) (control, intervention)
Arzola-Paniagua _(a)_ et al. [18]	2016	24/15	Obesity/Mexico	Resveratrlol 300 mg	24 weeks	TG	33.7 ± 11.9, 38.8 ± 9.59
Arzola-Paniagua _(b)_ et al. [18]	2016	21/24	Obesity/Mexico	Resveratrlol 300 mg	24 weeks	TG	40.96 ± 10.0, 39.76 ± 8.91
Bashmakov YK et al. [19]	2014	10/14	T2DM/Egypt	Resveratrol 100 mg	2 months	HDL-C, LDL-C, and TC	54 ± 10.1, 59.8 ± 6.6
Bhatt JK et al. [20]	2012	29/28	T2DM/India	Resveratrol 250 mg	3 months	TG, HDL-C, LDL-C, and TC	56.67 ± 8.91, 57.75 ± 8.71
Goh KP et al. [21]	2014	5/5	T2DM/Singapore	Resveratrol 3000 mg	12 weeks	TG, HDL-C, LDL-C, and ALT	55.8 ± 7.3, 56.8 ± 5.3
Imamura H et al. [22]	2017	25/25	T2DM/Japan	Resveratrol 100 mg	12 weeks	TG, HDL-C, TC	57.4 ± 10.6, 58.2 ± 10.1
Zare Javid A et al. [23]	2017	22/21	T2DM/Iran	Resveratrol 480 mg	4 weeks	TG	49.1 ± 7.4, 50.9 ± 8.9
Kjær TN et al. [24] _(a)_	2017	12/21	MetS/Denmark	Resveratrol 150 mg	16 weeks	TG, HDL-C, LDL-C, TC, and ALT	49.1 ± 6.69, 47.8 ± 6.36
Kjær TN et al. [24] _(b)_	2017	12/21	MetS/Denmark	Resveratrol 1000 mg	16 weeks	TG, HDL-C, LDL-C, TC, and ALT	51.9 ± 5.86, 47.8 ± 6.36
Kumar BJ et al. [25]	2013	29/28	T2DM/India	Resveratrol 250 mg	6 months	TG, HDL-C, LDL-C, and TC	56.67 ± 8.91, 57.75 ± 8.71
Militaru C et al. [26] _(a)_	2013	29/29	Stable angina/Romania	Resveratrol 20 mg	2 months	TG, HDL-C, LDL-C, and TC	64.9 ± 5.8, 64.2 ± 7.1
Militaru C et al. [26] _(b)_	2013	29/29	Stable angina/Romania	Resveratrol 20 mg	2 months	TG, HDL-C, LDL-C, and TC	66.3 ± 5.5, 63.7 ± 6.2
Most J et al. [27]	2016	20/18	Obese/Netherlands	Resveratrol 80 mg + 282 mg epigallocatechin-3-gallate	12 weeks	TG, HDL-C, LDL-C, and TC	36.1 ± 9.33, 38.7 ± 9.83
Movahed A et al. [28]	2013	31/33	T2DM/Iran	Resveratrol 1000 mg	45 days	TG, HDL-C, LDL-C, TC, ALT,AST, and GGT	52.45 ± 6.18, 51.81 ± 6.99
Poulsen MM et al. [29]	2013	12/12	Obese/ Denmark	Resveratrol 1500 mg	4 weeks	TG, HDL-C, LDL-C, TC, and ALT	44.7 ± 12.12, 31.9 ± 10.03
Seyyedebrahimi S et al. [30]	2018	23/23	T2DM/ Iran	Resveratrol 800 mg	2 months	TG, HDL-C, LDL-C, TC, ALT, and AST	54.96 ± 6.37, 58.72 ± 6.06
Méndez-del Villar M et al. [31]	2014	10/11	MetS/ Mexico	Resveratrol 1500 mg	3 months	TG, HDL-C, LDL-C, and TC	39.8 ± 5.4, 40.3 ± 5.4
Witte AV et al. [32]	2014	23/23	Overweight subjects/Germany	Resveratrol 200 mg	26 weeks	TG and TC	64.8 ± 6.8, 63.7 ± 5.3
Chachay VS et al. [33]	2014	10/10	NAFLD/Australia	Resveratrol 3000 mg	8 weeks	TG, HDL-C, LDL-C, TC, ALT, and AST	48.8 ± 12.2, 47.5 ± 11.2
Chen S et al. [34]	2015	30/30	NAFLD/China	Resveratrol 300 mg	12 weeks	TG, HDL-C, LDL-C, TC, ALT, AST, and GGT	45.2 ± 10.0, 43.5 ± 11.0
Faghihzadeh F et al. [35]	2015	25/25	NAFLD/Iran	Resveratrol 500 mg	12 weeks	TG, HDL-C, LDL-C, TC, ALT, AST, and GGT	44.04 ± 10.10, 46.28 ± 9.52
Kantartzis K et al. [36]	2018	52/53	Overweight and insulin resistant Subjects/Germany	Resveratrol 150 mg	12 weeks	TG, HDL-C, LDL-C, TC, ALT, AST, and GGT	18–70
Most J et al. [37]	2018	14/11	Obesity/Netherlands	Resveratrol 80 mg + 282 mg epigallocatechin-3-gallate	12 weeks	TG	36 ± 3, 40 ± 3
Khodabandehloo H et al. [38]	2018	20/25	T2DM/Iran	Resveratrol 800 mg/day	8 weeks	TG, HDL-C, LDL-C, TC, ALT, and AST	56.48 ± 6.72, 61.10 ± 5.61
Chekalina NL et al. [39]	2016	33/30	CAD/Ukraine	Resveratrol 100 mg	2 months	TG, HDL-C, LDL-C, and TC	48–72
Fujitaka K et al. [40]	2011	17/17	MetS/Japan	Trans resveratrol 100 mg (Longevinex)	3 months	TG, HDL-C, and LDL-C	63 ± 9, 62 ± 14
Cicero AF et al. [41]	2016	0verall 25	Hypercholesterolemic/Italy	Resveratrol 20 mg and monacolins from *M. purpureus *10 mg	4 weeks	TG, HDL-C, LDL-C, TC, ALT, and AST	18–70
Biesinger S et al. [42]	2016	Overall 18	Hypertension/USA	Resveratrol 60 mg	4 weeks	TG, HDL-C, LDL-C, and TC	44 ± 3
Timmers S et al. [43]	2011	Overall 11	Obesity/Netherlands	Resveratrol 150 mg	30 days	TG	52.5 ± 6.95, 52.5 ± 6.95
van der Made SM et al. [44]	2015	Overall 45	Obesity/Netherlands	Resveratrol 150 mg	4 weeks	HDL-C and TC	61 ± 7
de light M et al. [45]	2018	Overall 13	T2DM/Netherlands	Resveratrol 150 mg	30 days	HDL-C, LDL-C, TC, AST, and GGT	66 ± 7.7
Simental-Mendía LE et al. [46]	2019	31/31	Dyslipidemia/México	Resveratrol 100 mg	8 weeks	TG, HDL-C, LDL-C, and TC	20–65
Fodor K et al. [47]_(a)_	2018	46/81	Stroke/Romania	Resveratrol 100 mg + Allopathic treatment + physical rehabilitation	48 weeks	TG, HDL-C, LDL-C, and TC	65.03 ± 8.24, 64.78 ± 6.32
Fodor K et al. [47]_(b)_	2018	46/55	Stroke/Romania	Resveratrol 200 mg + Allopathic treatment + physical rehabilitation	48 weeks	TG, HDL-C, LDL-C, and TC	64.52 ± 8.05, 64.78 ± 6.32
Mazza A et al. [48]	2018	30/30	Hypertensive and hypercholesterolemic subjects/Italy	Nutraceutical compounds capsule containing resveratrol 20 mg + standardized Mediterranean diet	4 weeks	TG, HDL-C, LDL-C, TC, ALT, and AST	51.5 ± 7.8, 53.0 ± 8.1

*CAD* Coronary artery disease, *MetS* Metabolic syndrome, *NAFLD* Non-alcoholic fatty liver disease, *NR* Not reported, *T2DM* Type 2 diabetes mellitus, *LDL-C* Low-density lipoprotein-cholesterol, *HDL-C* High-density lipoprotein-cholesterol, *ALT* Alanine aminotransferase, *AST* Aspartate aminotransferase, *GGT* Gamma-glutamyl transferase

### Main outcomes

#### Effects of resveratrol supplementation on lipid profiles and liver enzymes

**Fig. 3:**
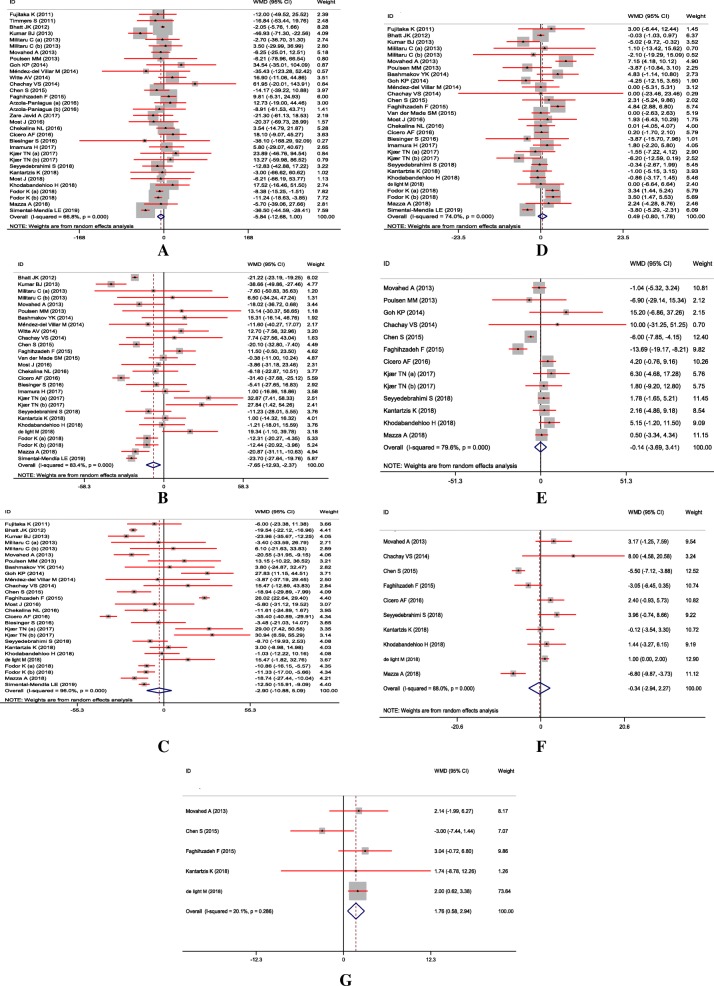
A-G Meta-analysis standardized mean differences estimates for (**a**) triglycerides (**b**) for total-, (**c**) for LDL-, (**d**) for HDL-cholesterol, (**e**) for ALT, (**f**) for AST, and (**g**) for GGT concentrations in the resveratrol and control groups (CI = 95%)

The impact of resveratrol supplementation on lipid profiles and liver enzymes are indicated in Fig. 3. The combined findings, using random-effects model showed that resveratrol intake significantly decreased total cholesterol (= − 7.65 mg/dL; 95% CI, − 12.93, − 2.37; *P* < 0.01; I^2^: 83.4%) and increased GGT concentrations (WMD = 1.76 U/l; 95% CI, 0.58, 2.94; *P* < 0.01; I^2^: 20.1%). We found no significant effect of resveratrol intake on triglycerides (WMD = -5.84 mg/dL; 95% CI, − 12.68, 1.00; *P* = 0.09; I^2^: 66.8%), LDL- (WMD = -2.90 mg/dL; 95% CI, − 10.88, 5.09; *P* = 0.47; I^2^: 96.0%), HDL-cholesterol (WMD = 0.49 mg/dL; 95% CI, − 0.80, 1.78; *P* = 0.45; I^2^: 74.0%), ALT (WMD = -0.14 U/l; 95% CI, − 3.69, 3.41; *P* = 0.93; I^2^: 79.6%), and AST (WMD = -0.34 U/l; 95% CI, − 2.94, 2.27; *P* = 0.80; I^2^: 88.0%) concentrations.

#### Subgroup analyses

The findings of subgroup analyses indicated that there were no significant changes between before and after subgroup analyses combined WMDs for lipid profiles and liver enzymes. The results of subgroup analyses are indicated in Table 2.

**Table 2:** The association between resveratrol intake on lipid profiles and liver enzymes using subgroup analysis

Variables		Number of WMD included	Subgroups	Pooled WMD (random effect)	95% CI	I^2^ (%)	Overall I^2^ (%)
Triglycerides		32	Overall	-5.84	−12.68, 1.00	66.8	66.8
	Type of intervention	6	Resveratrol plus other nutrients or drugs	−8.81	−13.67, − 3.96	0.0	
		26	Resveratrol	−5.06	− 14.41, 4.29	71.8	
	Dosage of resveratrol (mg/day)	10	≥ 500 mg resveratrol	1.43	−7.84, 10.69	0.0	
		22	< 500 mg resveratrol	−8.07	−16.20, 0.05	74.1	
	Duration of study (week)	14	< 12 weeks	−4.93	−19.17, 9.31	70.8	
		18	≥ 12 weeks	−5.40	−11.53, 0.74	40.5	
	Type of disease	8	Overweight or obese	1.44	−13.46, 16.34	0.0	
		16	Other	−5.41	−15.72, 4.91	74.1	
		8	T2DM	−8.06	−21.30, 5.17	58.4	
Total cholesterol		28	Overall	−7.65	−12.93, − 2.37	83.4	83.4
	Type of intervention	5	Resveratrol plus other nutrients or drugs	−18.10	−27.80, −8.39	80.5	
		23	Resveratrol	−4.02	−10.53, 2.49	84.4	
	Dosage of resveratrol (mg/day)	9	≥ 500 mg resveratrol	−2.47	−13.62, 8.67	64.3	
		19	< 500 mg resveratrol	−10.07	−15.81, −4.33	84.2	
	Duration of study (week)	15	< 12 weeks	−8.61	−16.64, −0.57	78.5	
		13	≥ 12 weeks	−5.29	−14.06, 3.49	87.6	
	Type of disease	5	Overweight or obese	1.93	−5.65, 9.50	0.0	
		15	Other	−8.32	−16.28, −0.36	83.8	
		8	T2DM	−9.68	−21.12, 1.76	83.4	
LDL-cholesterol		27	Overall	−2.90	−10.88, 5.09	96.0	96.0
	Type of intervention	5	Resveratrol plus other nutrients or drugs	−17.61	−29.20, −6.01	92.2	
		22	Resveratrol	0.84	−8.72, 10.39	96.1	
	Dosage of resveratrol (mg/day)	10	≥ 500 mg resveratrol	5.47	−9.90, 20.84	93.8	
		17	< 500 mg resveratrol	−9.89	−15.45, −4.33	86.2	
	Duration of study (week)	14	< 12 weeks	−7.38	−15.47, 0.71	85.9	
		13	≥ 12 weeks	0.59	−12.65, 13.84	97.6	
	Type of disease	3	Overweight or obese	3.47	−6.35, 13.29	0.0	
		16	Other	−2.94	−14.71, 8.82	97.0	
		8	T2DM	−5.10	−16.01, 5.80	88.1	
HDL-cholesterol		29	Overall	0.49	−0.80, 1.78	74.0	74.0
	Type of intervention	5	Resveratrol plus other nutrients or drugs	2.31	0.63, 3.98	44.4	
		24	Resveratrol	−0.02	−1.52, 1.49	73.4	
	Dosage of resveratrol (mg/day)	10	≥ 500 mg resveratrol	0.55	−2.21, 3.32	78.2	
		19	< 500 mg resveratrol	0.31	−1.11, 1.72	69.1	
	Duration of study (week)	15	< 12 weeks	0.26	−1.66, 2.18	72.1	
		14	≥ 12 weeks	0.79	− 0.92, 2.50	71.1	
	Type of disease	4	Overweight or obese	−0.46	−2.51, 1.59	0.0	
		16	Other	0.61	−1.55, 2.76	69.3	
		9	T2DM	0.63	−1.43, 2.70	74.3	
ALT		13	Overall	−0.14	−3.69, 3.41	79.6	79.6
	Type of intervention	2	Resveratrol plus other nutrients or drugs	2.00	−1.56, 5.56	25.2	
		11	Resveratrol	−0.74	−4.88, 3.41	78.9	
	Dosage of resveratrol (mg/day)	9	≥ 500 mg resveratrol	−1.77	−6.37, 2.84	80.8	
		4	< 500 mg resveratrol	2.19	−0.51, 4.89	0.0	
	Duration of study (week)	7	< 12 weeks	1.52	−0.39, 3.43	0.0	
		11	≥ 12 weeks	−2.01	−8.09, 4.08	78.2	
	Type of disease	2	Overweight or obese	1.34	−5.36, 8.04	0.0	
		7	Other	−1.76	−7.00, 3.48	83.8	
		4	T2DM	1.75	−1.43, 4.92	27.3	
AST		10	Overall	−0.34	−2.94, 2.27	88.0	88.0
	Type of intervention	2	Resveratrol plus other nutrients or drugs	−2.22	−11.24, 6.79	93.7	
		8	Resveratrol	0.18	−2.70, 3.05	87.5	
	Dosage of resveratrol (mg/day)	6	≥ 500 mg resveratrol	0.28	−3.78, 4.35	84.1	
		4	< 500 mg resveratrol	−0.83	−4.42, 2.76	87.5	
	Duration of study (week)	7	< 12 weeks	0.96	−1.92, 3.84	79.1	
		3	≥ 12 weeks	−3.16	−6.43, 0.12	76.1	
	Type of disease	1	Overweight or obese	−0.12	−3.54, 3.30	–	
		5	Other	−2.61	−6.29, 1.06	83.6	
		4	T2DM	1.23	0.30, 2.17	0.0	
GGT		5	Overall	1.76	0.58, 2.94	20.1	20.1
	Type of intervention	–	Resveratrol plus other nutrients or drugs	–	–	–	
		5	Resveratrol	1.76	0.58, 2.94	20.1	
	Dosage of resveratrol (mg/day)	3	≥ 500 mg resveratrol	1.05	−1.31, 3.40	55.9	
		2	< 500 mg resveratrol	2.00	0.63, 3.36	0.0	
	Duration of study (week)	2	< 12 weeks	2.01	0.71, 3.32	0.0	
		3	≥ 12 weeks	0.60	−2.17, 3.37	52.2	
	Type of disease	1	Overweight or obese	1.74	−8.78, 12.26	–	
		2	Other	0.52	−2.35, 3.39	75.8	
		2	T2DM	2.00	0.71, 3.32	0.0	

*ALT* Alanine aminotransferase, *AST* Aspartate aminotransferase, *GGT* Gamma-glutamyl transferase

#### Sensitivity analyses

Sensitivity analyses showed no significant changes between the pre- and post-sensitivity combined WMDs for triglycerides, HDL-cholesterol, ALT, AST, and GGT concentrations. We found that there were a significant effect between before and after sensitivity pooled WMD for total cholesterol after removing Bhatt et al. [20] study (WMD -5.76; 95% CI, − 12.23, 0.70), and for LDL-cholesterol after removing Faghihzadeh et al. [35] study (WMD -6.32; 95% CI, − 11.41, − 1.22) (Table 3).

**Table 3:** The association between resveratrol intake and lipid profiles and liver enzymes using sensitivity analyses

Variables	Pre-sensitivity analysis			Upper & lower of effect size	Post-sensitivity analysis		
	No. of studies included	Pooled WMD (random effect)	95% CI		Pooled WMD (random effect)	95% CI	Excluded studies
Triglycerides	32	−5.84	−12.68, 1.00	Upper	−4.24	−8.78, 0.29	Simental-Mendía [46]
				Lower	−6.86	−13.88, 0.15	Faghihzadeh [35]
Total cholesterol	28	−7.65	−12.93, −2.37	Upper	−5.76	− 12.23, 0.70	Bhatt [20]
				Lower	−9.00	−14.11, −3.89	Faghihzadeh [35]
LDL-cholesterol	27	− 2.90	−10.88, 5.09	Upper	−1.49	−9.36, 6.37	Cicero [41]
				Lower	−6.32	−11.41, − 1.22	Faghihzadeh [35]
HDL-cholesterol	29	0.49	−0.80, 1.78	Upper	0.69	−0.59, 1.99	Kumar [25]
				Lower	0.17	−1.04, 1.40	Movahed [28]
ALT	13	− 0.14	− 3.69, 3.41	Upper	− 0.33	− 4.10, 3.42	Kantartzis [36]
				Lower	− 0.69	−4.35, 2.96	Khodabandehloo [38]
AST	10	−0.34	− 2.94, 2.27	Upper	0.42	− 2.19, 3.03	Mazza [48]
				Lower	−0.77	−3.51, 1.96	Seyyedebrahimi [30]
GGT	5	1.76	0.58, 2.94	Upper	2.11	0.89, 3.34	Chen [34]
				Lower	1.07	−1.22, 3.37	de Light M [45]

*ALT* Alanine aminotransferase, *AST* Aspartate aminotransferase, *GGT* Gamma-glutamyl transferase

#### Publication bias and quality assessment

Egger and Begg’s tests indicated no significant effect of possible publication bias for meta-analyses calculating the influence of resveratrol intake on triglycerides (P Begg’s test = 0.74, P Egger’s test = 0.69), LDL-cholesterol (P_Bg_ = 0.07, P_Ee_ = 0.53), HDL-cholesterol (P_Bg_ = 0.88, P_Ee_ = 0.98), ALT (P_Bg_ = 0.39, P_Ee_ = 0.11), AST (P_Bg_ = 0.42, P_Ee_ = 0.90), and GGT concentrations (P_Bg_ = 0.14, P_Ee_ = 0.60). The authors found that there was a significant effect of the potential of publication bias for total-cholesterol (P_Bg_ = 0.17, P_Ee_ = 0.00). We applied non-parametric method (Duval and Tweedie) to calculate the findings of censored articles for total-cholesterol; however, pooled WMDs findings did not statistically significant change after using Duval and Tweedie test.

## Discussion

The findings of current systematic review and meta-analysis showed that resveratrol supplementation among patients with MetS and related disorders significantly reduced total cholesterol and increased GGT concentrations, but did not affect triglycerides, LDL-, HDL-cholesterol, ALT, and AST concentrations.

MetS and related disorders are characterized by changes in fatty acid metabolism, which finally results in decreased HDL-cholesterol, and increased LDL-cholesterol as well as, triglycerides concentrations. As dyslipidemia is a well-established risk factor for MetS, diabetes, and CVDs, circulating lipid profiles are routinely addressed by pharmacotherapy. We found that resveratrol supplementation among patients with MetS and related disorders significantly reduced total cholesterol, but did not affect triglycerides, LDL-, HDL-cholesterol concentrations. Previously, the effects of resveratrol on weight loss [49] and biomarkers of inflammation and oxidative stress among patients with MetS [50], and coenzyme Q10 on lipid profiles among patients diagnosed with coronary artery disease [51] were assessed. In a study conducted by Simental-Mendia et al. [46], resveratrol supplementation at a dosage of 100 mg/day for 8 weeks to individuals with dyslipidemia significantly decreased total cholesterol and triglycerides concentrations. In addition, taking resveratrol supplements at a dosage of 300 mg/day for 3 months by patients with non-alcoholic fatty liver disease significantly decreased total- and LDL-cholesterol concentrations [34]. The supplementation of resveratrol plus D-chiro-inositol for 60 days among overweight pregnant woman with an increased fasting glucose significantly reduced total-, LDL-cholesterol, and triglycerides concentrations [52]. However, in a meta-analysis conducted by Zhang et al. [53], resveratrol supplementation significantly increased total- and LDL-cholesterol concentrations. In addition, another meta-analysis found no significant effects on lipid variables following the supplementation of resveratrol in patients with T2DM [16]. The hypocholesterolemic effect of resveratrol may be mediated by its phenolic hydroxyls contain that lead to oxidation of the unsaturated fatty acids and decreasing circulating cholesterol [54]. In addition to the beneficial effects of resveratrol on lipid metabolism, the anti-atherosclerotic activity of resveratrol involves enhanced activity of peroxisome proliferator-activated receptor α [55], suppressing platelet aggregation [56], reduced blood pressure [43], and improvement of the endothelial activity [57]. Therefore, it is expected that resveratrol administration among patients with MetS and related disorders exerts a potential cardioprotective impact.

The current meta-analysis demonstrated that taking resveratrol supplements by patients with MetS and related disorders was associated with a significant reduction in GGT, but did not affect ALT and AST concentrations. In a study by Asghari et al. [58], resveratrol supplementation at a dosage of 600 mg/day for 12 weeks to patients with NAFLD did not modify liver enzymes and oxidative/anti-oxidative status. In addition, previous animal studies have claimed that resveratrol protects the liver against steatosis [59] and decreases intracellular lipids in the liver [60]. In another study, Heebøll et al. [61] demonstrated no significant improvement in the intrahepatic lipid content and the circulating concentrations of liver enzymes following resveratrol supplementation at a dosage of 1500 mg/day for 6 months among patients with NAFLD. An 8-week resveratrol supplementation at a dosage of 3000 mg/day, not only failed to show any significant improvements in NAFLD features, but also significantly increased liver enzymes concentrations [33]. Also, Faghihzadeh et al. [62] demonstrated that 500 mg/day resveratrol supplementation for 3 months among people with NAFLD significantly improved liver steatosis and ALT concentrations. A similar study with 600 mg/day resveratrol also documented a significant improvement in liver enzymes concentrations without any changes in liver steatosis degree [34]. These inconsistent findings could be related to the stage of disease, type of diseases, the method of measuring liver fat content, different dosage of resveratrol used, or baseline metabolic characteristics of the participants.

## Conclusions

This meta-analysis demonstrated that resveratrol supplementation to the patients with MetS and related disorders significantly reduced total cholesterol and increased GGT concentrations, but did not affect triglycerides, LDL-, HDL-cholesterol, ALT, and AST concentrations. Therefore, resveratrol supplementation to patients with MetS and related disorders may have a potential cardio-protective effect through the reduction of total cholesterol and GGT concentrations.

## Data Availability

The primary data for this study is available from the authors on direct request.

## Abbreviations

ALT: Alanine aminotransferase
AST: Aspartate aminotransferase
CADs: Coronary artery diseases
GGT: Gamma-glutamyl transferase
HDL-C: High-density lipoprotein-cholesterol
LDL-C: Low-density lipoprotein-cholesterol
MetS: Metabolic syndrome
NAFLD: Non-alcoholic fatty liver disease
NR: Not reported
T2DM: Type 2 diabetes mellitus
